# Multi-Target Approaches in Colon Cancer Chemoprevention Based on Systems Biology of Tumor Cell-Signaling

**DOI:** 10.4137/grsb.s486

**Published:** 2008-05-02

**Authors:** Suresh Guruswamy, Chinthalapally V. Rao

**Affiliations:** Department of Medicine, Hematology-Oncology Section, University of Oklahoma Health Sciences, Center, Oklahoma City, OK, U.S.A

**Keywords:** colon cancer, chemoprevention, COX-2, HMG-CoA reductase, caveolae

## Abstract

Colorectal cancer is the leading cause of cancer related deaths in the United States. Although it is preventable, thousands of lives are lost each year in the U.S. to colorectal cancer than to breast cancer and AIDS combined. In colon cancer, the formation and progression of precancerous lesions like aberrant crypt foci and polyps is associated with the up-regulation of cycloxygenase-2 (COX-2), inducible nitric oxide synthase (iNOS) and hydroxy methyl glutaryl CoA reductase (HMG-CoA reductase). The current review will focus on the signaling pathway involving COX-2 and HMG-CoA reductase enzymes and their downstream effectors in signaling mechanism. Cancer cells need huge pools of both cholesterol and isoprenoids to sustain their unlimited growth potential. Cholesterol by modulating caveolae formation regulates several signaling molecules like AKT, IGFR, EGFR and Rho which are involved in cell growth and survival. Cholesterol is also essential for lipid body formation which serves as storage sites for COX-2, eicosanoids and caveolin-1. Experimental studies have identified important mechanisms showing that COX-2, caveolin-1, lipid bodies and prenylated proteins is involved in carcinogenesis. Therefore multi-target, multi-drug approach is the ideal choice for effective colon cancer chemoprevention. This review will give an overview of the two pathways, their signaling networks, and the interactions between the components of the two networks in the activation and regulation of cell signaling involving growth/survival and explain the rationale for colon cancer chemoprevention using COX-2 inhibitors and statins.

## Introduction

The systems biology approach to gene regulation involves the study of networks of genes between well-characterized pathways. However, the fundamental challenge is to predict how a certain drug interacts with the cellular and molecular components of the signaling network. This will help in understanding the expected behavior of a drug interaction with the signaling network. The interaction will then guide the behavior of the cell. Therefore, the identification of the signaling network or crosstalk among various signal pathways is of fundamental importance in developing novel strategies for prevention and treatment of cancer. Comparative analysis of these networks is central to our understanding of the biological systems.

Today, our knowledge and technology have reached a point where it is possible to identify individuals at risk for cancer and disrupt the processes at the level of the pre-cancerous lesion thereby blocking the neoplastic transformation and ultimately metastasis. Understanding the systems biology of colonic pre-neoplasia and progression to adenoma and adenocarcinoma enhances the likelihood of designing novel chemoprevention strategies for prevention of colon cancer. The current review will focus on targeting multiple pathways for colon cancer chemoprevention. The underlying basis of many cancers is due to defective regulation of signaling pathways that control cellular proliferation and differentiation. It is now thought that normal self-renewing stem cells that persist throughout life become tumorigenic as a result of accumulated mutations and the transforming events that lead to dysregulation of their metabolic pathways. Understanding the differences between the cross-talk that occurs in normal cells, and the cross-talk that occurs in cancer cells will provide insight that could lead to new strategies for treating cancer.

## Significance of Colorectal Cancer

Colorectal cancer is one of the leading causes of cancer related death in the western world, including the United States. The American Cancer Society estimates that about 112,340 new cases of colon cancer (55,290 men and 57,050 women) and 41,420 new cases of rectal cancer (23,840 men and 17,580 women) will be diagnosed in the United States in 2007. It is the second leading cause of cancer-related deaths with 52,180 deaths (26,000 men and 26,180 women) expected during 2007 (American Cancer Society, 2007). The costs from hospitalization are much more among colorectal cancer patients than among lung, prostrate and breast cancer patients. Most colorectal cancers start as non-cancerous polyps, some of which progress in size to form large polyps (>1.0 cm), and then to adenoma and carcinoma (Janne and Mayer, 2000, Fig. 1). Since colorectal cancers develop slowly over a long period, which is at least ten years in most people (Smith et al. 2001), chemoprevention can be used to control colon cancer progression. The data from human and animal models show that the etiology of colon cancer is multifactorial and complex. As a result, increasing efforts are being focused on developing more effective screening and prevention measures for colorectal cancer. A major area of research emphasis is the inverse relationship between certain biochemical pathways and the development of cancer. A number of metabolic pathways or individual molecules are upregulated in colon cancer. The current review will focus on the upregulation of COX-2 and HMG-CoA reductase enzymes and the signaling cascades that are altered as a result of this up-regulation.

Under normal physiologic circumstances, COX-2 is expressed in the GI tract at undetectable levels. But, this expression rises significantly in inflammation and colorectal malignancies (Kutchera et al. 1996; Kargman et al. 1996). For example, COX-2 is up-regulated 2 to 50-fold in 85% to 90% of colorectal tumors, making the COX-2 enzyme a good target candidate for chemoprevention (Eberhart CE, 1994). The observation that HMG-CoA reductase is upregulated in colon cancer came from two large clinical trials designed to study the changes in coronary events in coronary heart disease. In patients receiving HMG-CoA reductase inhibitors (pravastatin, simvastatin), the number of newly diagnosed colon cancer cases reduced by 43% and 19% during a 5-year follow-up period (Sacks et al. 1996; Pedersen et al. 1996). A thorough understanding of the differences between altered signaling pathways in cancer cells and the signaling pathways in normal cells will lead to the design of new therapeutic strategies for suppressing the tumorigenic and invasive potential of malignant tumors.

## COX-2 and Colon Cancer

COX is a bifunctional enzyme that sequentially catalyzes cyclooxygenase and peroxidase reactions (Smith et al. 1991). It is a membrane-bound heme-protein that catalyzes the bisoxygenation of arachidonic acid (a fatty substance) to form prostaglandin PGG_2_ and the peroxidative reduction of PGG_2_ to form prostaglandin PGH_2_ (Smith et al. 1991). Prostaglandins are hormone-like substances found in very small quantities in the body which have potent physiological effects. There are two structural forms of cyclooxygenase, COX-1 (Cycloxygenase 1) and COX-2 (Smith et al. 2000). COX-1 was identified in 1976 (Hemler et al. 1976; Miyamoto et al. 1976) and cloned a decade later by three different groups (Merlie et al. 1988; DeWitt, 1988; Yokoyama et al. 1989). Cyclooxygenase-1 (COX-1) is an enzyme which is normally present in a several areas of the body, including sites of inflammation and the stomach. COX-1 primarily serves a maintenance function with stable levels, while the gene for COX-2 is inducible, or stimulated by growth factors, cyto-kines, carcinogens, oncogenes and other tumor promoters. The COX-1 enzyme of the stomach produces prostaglandins that ensure the natural mucus lining of the stomach and the integrity of the blood platelets. Prostaglandins synthesized by the COX-1 enzyme are responsible for maintenance and protection of the gastrointestinal tract, while prostaglandins synthesized by the COX-2 enzyme are responsible for inflammation and pain (Smith and Dewitt, 1996; Williams et al. 1996a). When the COX enzymes are blocked, inflammation is reduced, but the protective mucus lining of the stomach is also reduced, which can cause stomach upset, ulceration and bleeding from the stomach and intestines.

In 1991, an inducible isoform of COX which was subsequently named COX-2 was identified using mitogen-stimulated chicken fibroblasts (Xie et al. 1991), phorbol-ester (Kujubu et al. 1991) and serum-stimulated murine fibroblasts (O’Banion et al. 1991). The COX-2 enzyme is localized in areas of the body that are responsible for inflammation and not in the stomach. Since the COX-2 enzyme does not play a role in the normal function of the stomach or intestinal tract, medications which selectively block COX-2 do not present the same risk of injuring the stomach or intestines. The relationship between cancer and inflammation due to chronic infection has been suspected, but not proven, for many years. In 1972, Haddow showed the similarities between wound healing and carcinogenesis (Haddow A, 1972). Subsequently, Dvorak described tumors as wounds that do not heal (Dvorak, 1986). In addition to colon cancer, COX-2 overexpression has also been demonstrated in a number of premalignant and malignant conditions including non-small-cell lung cancer (NSCLC), Barrett’s esophagus and Barrett’s adenocarcinomas (Wilson, 1998) and head and neck cancer (Chan et al. 1999).

Compelling evidence for the role of COX-2 in the formation of colorectal cancers has been provided by genetic studies in mice. COX-2 levels are markedly increased in human colorectal adenocar-cinomas and intestinal tumors that develop in carcinogentreated rats and APC Min mice (Kargman et al. 1995; DuBois et al. 1996; Williams et al. 1996b). Direct evidence that COX-2 is involved in colon cancer came from studies with knockout mice for COX-2. The study in mice showed that COX-2 knockout reduced both the size and number of intestinal polyps (Oshima et al. 1996). In a model of mammary tumorigenesis, overexpression of COX-2 alone in mammary glands was sufficient to induce cellular transformation which resulted in the formation of breast carcinomas (Liu et al. 2001).

The reason for the overexpression of COX-2 in cancer has not been exactly determined, because contrary to many other genes, the gene that codes for this enzyme has no mutations. COX-2 plays a role in angiogenesis, the growth of small blood vessels that allows tumors to continue to grow (Jones et al. 1999). Most of the actions attributed to COX-2 seem to be exerted by the metabolic products of the COX-2 pathway, prostaglandins. Prostaglandins synergize with mediators like histamine to elicit enhanced vascular permeability (Tilley et al. 2001). Another important action of the COX-2 enzyme is inhibition of apoptosis, which in many cases constitutes another mechanism to promote tumor cell growth. Overexpression of COX-2 is associated with increased proliferation and decrease in apoptosis (Tsujii and Dubois, 1995). The decreased cell death caused by COX-2 and PGE_2_ enhances the tumorigenic potential of intestinal epithelial cells (Sheng, 1998). In another study, stromal production of prostacyclin was shown to confer an antiapoptotic effect to colonic epithelial cells (Cutler et al. 2003). Constitutive expression of COX-2 can also lead to phenotypic changes which alter the metastatic potential of colorectal cancer. Biochemical changes associated with this phenotypic change includes the activation of membrane-type metalloproteinase-2 (Tsujii et al. 1997).

## Mechanism of COX-2 Action in Cancer

The exact molecular mechanism of how COX-2 causes cancer is yet to be understood. A number of studies suggest that the products of the COX pathway, prostaglandins, especially the PGE_2_, enhance cell proliferation in both normal and cancer cells through specific prostaglandin receptors (EP) (Yano T et al. 2002; Pai et al. 2001; Pai et al. 2002). The EP receptors are a family within the G protein-coupled receptor (GPCR) superfamily of seven-transmembrane spanning proteins. There are at least nine different prostaglandin receptor forms reported till date (Narumiya and Fitzgerald, 2001). Four of these receptor subtypes bind PGE_2_ (EP_1_-EP_4_), two bind PGD_2_ (DP_1_ and DP_2_), and the other receptors bind PGF_2_, PGI_2_, and TxA2 (FP, IP, and TP) respectively (Narumiya et al. 1999; Breyer et al. 2001; Hirai et al. 2001). All EP receptors are localized on the plasma membrane with the exception of the prostaglandin E_2_ receptor that is localized on the nuclear membrane (Bhattacharya et al. 1998). Although prostaglandins have been implicated in functions in practically every organ, tissue and cell in the human body, the most prominent area of interest is its role in pain and inflammation. As more reports emerge linking inflammation to colon cancer, more studies are being carried out to study the effect of prostaglandins on cancer. Disrupted gene targeting, such as knockouts, have provided valuable information into the role of prostaglandins and their receptors. PGE_2_ is widely considered to be the most important prostaglandin with regard to colorectal carcinogenesis. In colon cancer, large amounts of PGE_2_ are formed with levels rising dramatically as cells progress to an invasive phenotype (Pugh and Thomas, 1994; Rigas et al. 1993). A knockout of the prostaglandin receptor EP_2_ gene reduces both the size and number of intestinal tumors in APC^716^ mice, similar to deficiency of the COX-2 gene (Sonoshita et al. 2001). Akt (protein kinase B), a serine/threonine kinase, is as a critical enzyme in signal transduction pathways involved in cell proliferation, apoptosis and angiogenesis. Akt plays a key role in the regulation of cell adhesion and actin rearrangement (Osada et al. 1999) and PGE_2_ stimulation of colon cancer cell growth has been shown to activate protein kinase AKT (Castellone et al. 2005; Sheng et al. 2001). PGE_2_ is also involved in maintaining the stability and activation of β-catenin. Cytoplasmic β-catenin is involved in signal transduction and activation of several genes like uPAR, *c-my*c, gastrin, COX-2, MMP-7 and cyclin D1, all of which play important roles in the development and progression of colorectal carcinoma (Alexander, 2002). PGE_2_ increases colon cancer cell invasiveness by transactivating c-Met-R, which in turn increases tyrosine phosphorylation of β-catenin. The stabilized β-catenin translocates to the nucleus and triggers uPAR, a target gene of β-catenin, which plays a major role in cell migration and invasiveness (Pai et al. 2003; Andreasen et al. 1997). Interestingly, u-PAR mRNA is overexpressed in cells located at the tumoral-stromal interface of invasive foci of human colon adenocarcinomas (Pyke et al. 1991). Thus, PGE_2_ through the β-catenin-uPAR signaling pathway influences the proteolytic events occurring at the interface between stroma and malignant cells in human colon adenocarcinomas.

**Figure 1:**
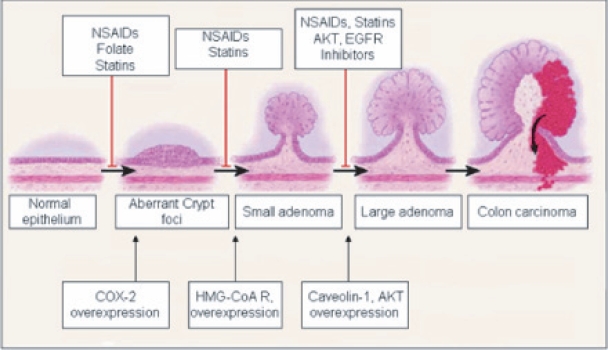
Colon cancer progression and overexpression of stage specific markers Colon cancer are originated from hyperproliferative regions in the normal colonic mucosa as polyps that develop into adenoma and finally carcinoma. The adenoma-carcinoma sequence is characterized by overexpression of several markers like COX-2, HMG-CoA-R, AKT and caveolin-1. Intervention of colon cancer progression can be achieved by using various chemopreventive agents either alone or in combination (modified from Janne and Mayer. 2000. New Engl J Med.)

NFκB (Nuclear factor κB) is another important signaling molecule whose activation induces the expression of COX-2, which leads to tissue inflammation (Yamamoto et al. 1995). Interestingly, the expression of the COX-2 gene is transcriptionally regulated by NFκB (D’Acquisto et al. 2002). Cancer cells have high levels of activated NFκB, which suggests that it plays an important role in malignancy. NFκB is normally present in the cytoplasm in an inactive state, bound to its inhibitor, IkB. Inflammatory stimuli release NFκB from the inhibitor, allowing it to translocate to the nucleus and regulate proteins that activate the genes responsible for growth, survival and the pathogenesis of the inflammatory process. Some of the genes that are directly activated by NFκB include the cellular inhibitors of apoptosis, such as c-IAP1, c-IAP2 and IXAP, the TNF receptor associated factors TRAF1 and TRAF2 and the antiapoptotic Bcl-2 homologue Mcl-1,A1/Bfl-1(Wang et al. 1998; Wu et al. 1998). NFκB also induces the expression of cyclin D1, which helps in G1-to-S-phase progression by binding directly to its promoter(Guttridgeet al.1999).Inhibition of NFκB activation can therefore reduce cyclin D1 activity and subsequent phosphorylation of the retinoblastoma protein. NSAIDs and corticosteroids can inhibit the activation of NFκB at different points in the signaling pathway. The inhibitory effects of aspirin result from the inhibition of ATP-binding to IKKβ, which reduces the phosphorylation of I*κ*B*α* and thus prevents its degradation (Yin, 1998). Several NSAIDs have also been shown to inhibit NFκB activation. For example, ibuprofen inhibits NFκB activation, as well as COX-2 expression and PGE_2_ production in murine macrophages (Lo et al. 1998). Another NSAID, sulindac, has been shown to decrease IKKβ kinase activity and, thereby inhibit NFκB activation (Yamamoto et al. 1999). Although it is clear that NFκB induces COX-2 expression, it is not yet known if COX-2 also positively regulates NFκB levels. A number of kinases, such as AKT and MEK kinases, activate IKK. AKT activates p-21 activated kinase (PAK1) and that stimulates the nuclear translocation of the NFκB subunit, p65 (Frost et al. 2000; Tang et al. 2000). Neither COX-2 nor its metabolite PGE_2_ activates NFκB directly. Since COX-2 and PGE_2_ activate AKT, and AKT is known to play a major role in NFκB activation, we can assume that COX-2 exerts its effect on NFκB through AKT. In a different scenario, COX-2 may also be regulating the activity of NFκB through proteins other than AKT. In spite of the above described mechanisms, the complete picture of how COX-2 and its proinflammatory metabolite PGE_2_ enhance colon cancer progression remains poorly understood. The schematic representation of COX-2 signaling mechanism is shown in Figure 1.

Because COX-2 is responsible for the increased PGE_2_ production in cancer, the inhibition of COX-2 activity is critical for colon cancer chemoprevention. Non-steroidal anti-inflammatory drugs (NSAIDs) block COX enzymes and reduce prostaglandins throughout the body. As a consequence, inflammation, pain and fever are reduced. Several studies have showed that COX-2-specific and non-specific NSAIDs induce apoptosis in a number of different cancers like gastric and lung cancer cells (Cao et al. 2002; Lin et al. 2002; Howe et al. 2002). Since NSAIDs block both COX-1 and COX-2, prostaglandins produced by COX-1 are also blocked. Therefore, a relatively new class of drugs called selective COX-2 inhibitors, such as valde-coxib, celecoxib and rofecoxib were introduced which specifically block the COX-2 enzyme. Celecoxib has been shown to inhibit aberrant crypt foci (ACF) incidence and multiplicity in AOM induced mouse carcinogenesis model (Reddy et al. 1996), as well as tumor incidence, multiplicity and volume (Reddy et al. 2000). Blocking the COX-2 enzyme impedes the production of the prostaglandins which cause pain, inflammation and cancer.

## HMG-CoA Reductase and Cancer

HMG-CoA reductase (or 3-hydroxy-3-methyl-glutaryl-CoA reductase or HMGR) is the first enzyme of the HMG-CoA reductase pathway, the metabolic pathway that produces cholesterol. Cholesterol, a major constituent of the eukaryotic cell membranes regulates the physical state of the phospholipid bilayer, affects the activity of several membrane proteins and is the precursor for steroid hormones and bile acids. Cholesterol also plays a crucial role in the formation of membrane microdomains, such as lipid rafts and caveolae. In addition to synthesizing cholesterol, HMG-CoA reductase also produces a number of non-sterol products. One of the first products synthesized by the HMG-CoA reductase is mevalonate. Mevalonate is converted to farnesyl diphosphate, geranylgeranyl diphosphate which are two important isoprenoids essential for the post-translational modification and biological activity of diverse array of proteins that have roles in cell shape, motility, cell division and survival. Inhibition of HMG CoA reductase will suppress the synthesis of isoprenoid moieties required for the post-translational modification of several important proteins like Ras, Rho, and lamin B and therefore offers a novel target for cancer chemoprevention.

## Caveolae in Cancer Cell Signaling

A wide variety of mammalian cells have specialized plasma membrane microdomains that are characterized by their high content of spingolipids and cholesterol (Okamoto et al. 1998). These regions, called lipid domains, are more rigid than the rest of the cell membrane because of cholesterol. These cholesterol rich regions can be isolated from the rest of the plasma membrane because they are resistant to dispersion by non-ionic detergents and are commonly referred as caveolae. Caveolae are cholesterol rich flask shaped invaginations present on the plasma membrane. Caveolae is known to be involved in several cellular events, including cholesterol trafficking and coordinating signaling events (Uittenbogaard et al. 1998). Caveolae and lipid rafts may serve as a way to cross-link certain pathways in the microdomains of the plasma membrane. The major component of caveolae is the membrane protein caveolin. There are three kinds of caveolin, named caveolin-1, caveolin-2 and caveolin-3 (Smart et al. 1999). All three caveolins are structurally similar and have hairpin loops criss-crossing the membrane. Caveolin-1 is the major component molecule of caveolae (Rothberg et al. 1992) and plays an important role in tumorigenesis and cancer progression. While, the expression of caveolin-1 is elevated in a majority of the colon adenocarcinomas, the expression of caveolin-2 is undetectable in either normal colonocytes, adenomas, or carcinomas (Fine et al. 2001; Patlolla et al. 2004). Caveolin-3 is not expressed in the colon and is restricted to muscular tissue (Tang et al. 1996). The identification of caveolins as the major proteins of caveolae has sparked research into the role and functions of caveolae. Caveolin-1 plays a critical role in organizing signaling machinery at the cell surface. The correct arrangement of signaling molecules in these domains is vital for communication inside the cell and is dependent on proper levels of cholesterol. Caveolin-1 contains a scaffolding domain to which a number of signaling molecules like G-protein-coupled receptors, heterotrimeric G proteins, receptor tyrosine kinases, components of the Rasmitogen-activated protein (MAP) kinase pathway, Src-like kinases, protein kinase C (PKC), nitric oxide synthase (NOS), H-Ras and eNOS are bound and concentrated (Anderson, 1993; Anderson, 1998; Li et al. 1995; Huang et al. 1997). Caveolin-1 itself may positively or negatively regulate signaling either through direct or indirect protein—protein interactions with resident caveolae proteins. The 20-amino acid membrane proximal region of the cytosolic amino-terminal domain of caveolin-1 is sufficient to mediate these interactions (Couet et al. 1997).

Caveolae plays pivotal roles in intracellular signal transduction, angiogenesis and tumor invasion (Simons and Ikonen, 1997; Simons and Toomre, 2000). Tumor cell invasion is an extremely important factor for the formation of solid tumors and necessary for its spread to different organs. The high mortality associated with colorectal cancer is related to its ability to spread beyond the large intestine and invade distant sites. Angiogenesis is the formation of new capillaries characterized by the proliferation, migration and remodeling of endothelial cells and their progenitors. The progressive growth and spread of tumors has been shown to be angiogenesis-dependent and a role for caveolin-1 in this process has been implicated (Woodman et al. 2003). For example, overexpression of caveolin-1 significantly enhances endothelial cell differentiation into tube-like structures and downregulation of caveolin-1 protein levels using antisense oligonucleotides reduced the ability of the endothelial cells to form an organized network (Liu et al. 2002; Griffoni et al. 2000). Similarly, tumor angiogenesis was reduced in caveolin^−/−^ mice implanted with B16 melanoma cells (Woodman et al. 2003). There is a significantly higher percentage of tumor associated endothelial cells (TAEC) in cav-1 positive tumors than in cav-1 negative tumors (Yang et al. 2007). Caveolin-1 is also an important regulator of eNOS signaling in endothelial cells. NO plays a major role in angiogenesis (Takahashi et al. 1997) acting downstream of vascular endothelial growth factor (VEGF). Cav−/− mice do not develop a functional vasculature and the effects observed in Cav−/− mice are similar to the effects of the NOS inhibitor L-NAME administered to Cav+/+ mice (Sonveaux et al. 2004). These results suggest that caveolin-1 is important for the organization of a new capillary network.

The concentration of caveolae near the intercellular junctions suggests that certain junctional molecules may colocalize with caveolin-1. Caveolin-1 has been shown to play a role in signal transduction mediated by integrin signaling (Totsukawa et al. 2000; Giancotti et al. 1999). Cell migration depends on integrins that attach cells to their substratum and regulate the organization of the cytoskeleton. The tyrosine phosphorylated form of caveolin-1 colocalizes with focal adhesions, suggesting a role for caveolin-1 in migration. *α*_V_β_3_ integrins were identified to colocalize with PI3-K/caveolin-1 complexes (Sedding et al. 2005). The integrin-caveolin-1 complex triggers the mitogen-activated protein kinase pathway, which controls progression through the cell cycle. Caveolin-1 mediated integrin signaling thus plays an important role in organizing cytoskeleton, cell migration and proliferation.

The overexpression or the targeted disruption of caveolin-1 has provided significant insights into the roles of the caveolin-1 and caveolae. Caveolin-1 constitutes a key switch in tumor development through its association of various signaling molecules. Disruption of membrane microdomains, like caveolae, with drugs like statins which sequester cholesterol will affect several signaling pathways associated with caveolae. Statins by blocking cholesterol synthesis leads to the perturbation of the lipid phase of the cell membrane and thus enhances the potenticity of anticancer drugs. The membrane mediated action of statins through modulation of membrane fluidity is also likely to play an important role in anticancer action and its ability to reverse multidrug resistance.

## Cholesterol and Lipid Bodies

One important aspect of cholesterol regulation is intracellular cholesterol storage in lipid storage organelles, called lipid droplets or lipid bodies. Mammalian lipid bodies are spherical structures containing a mixture of triglycerides or cholesterol esters encased in a thin phospholipid membrane (Martin and Parton, 2006). They also contain a pool of proteins with a wide range of biochemical activities. Only a few of these proteins have been identified, and very little is known about their structural properties and functions. Lipid bodies shuffle components around the cell, store energy in the form of neutral lipids and possibly maintain the many membranes of the cell. High concentrations of lipid bodies develop in cells associated with inflammation reactions (Coimbra et al. 1971; Weinstein, 1980). Various cytokines and pro-inflammatory stimuli trigger the synthesis of COX-2 leading to increased formation of eicosanoids. There is increasing evidence that specific compartmentalization of eicosanoid formation within cells may relate to the different autocrine and paracrine functions of eicosanoids (Serhan, 1996; Smith et al. 1996). Lipid bodies are sites of intracellular localization of COX-2 (Dvorak et al. 1994; Bozza et al. 1997a), as well as repositories of esterified arachidonates (Weller et al. 1991). Lipid bodies serve as novel putative sites for eicosanoid biosynthesis in cells involved in inflammation (Bozza et al. 1996; Bozza et al. 1997b). Caveolin-1 plays an important role in the modulation of lipolysis and lipid droplet formation (Cohen et al. 2004). In support of this, Caveolin-1 has been shown to be redirected from the plasmamembrane caveolae to intracellular lipid droplets (Razani et al. 2002). Caveolin-1 is also important for maintaining the architecture of the lipid droplet cortex. For example, caveolin-1 null perigonadal adipocytes exhibit dramatically altered lipid droplet cortex compared to normal adipocytes (Cohen et al. 2004). Since lipid bodies contain cholesterol, COX-2 as well as caveolin-1, statins will be useful in disrupting the membrane integrity of the lipid bodies while COX-2 inhibitors can be used to block the activity of the COX-2 enzyme.

## Prenylation and Cell Signaling

Although the beneficial effects of statins result from their cholesterol lowering properties that lead to disruption of membrane microdomains and possibly lipid bodies, recent observations also suggest that statins exhibit effects independent of their cholesterol-lowering properties. In addition to synthesizing cholesterol, the HMG-CoA reductase pathway also produces several biological intermediates, called isoprenoids, which include geranyl pyrophosphate and farnesyl pyrophosphate. Iso-prenoids serve as important lipid attachments to the C-terminal cysteine’s of proteins such as small GTP binding proteins, which are implicated in intracellular signaling. Prenylated proteins play a central role in signaling an array of cellular responses, such as cell division, motility and apoptosis (Table 1). Prenylation reactions are catalyzed by protein farnesyltransferase (FTase) and protein geranylgeranyltransferases (GGTases), respectively. Prenylation promotes the membrane association of the target protein (Casey and Seabra, 1996). The covalent addition of an isoprenoid to the carboxyl terminal cysteine changes the polarity of the protein and enables its binding to membranes. The isoprenoid geranylgeranylpyrophosphate activates a group of Rho, Rac and cdc42 proteins (Adamson et al. 1992). These proteins exert a wide range of effects that include cell proliferation, migration and increasing oxidation stress. A few important examples of farnesylated proteins include Ras and lamin B. Tumor cells exhibit a greater need for isoprenoids than normal cells (Buchwald, 1992). Tumor cells are, therefore, associated with a sterol feedback resistant HMG-CoA reductase activity, which ensures a steady pool of sterologenic pathway intermediates (Elson et al. 1999).

The family of proteins known as Ras plays a central role in integrating the regulatory signals that govern the cell cycle and proliferation (White et al. 1995). Defects in the Ras-Raf pathway can result in cancerous growth. Mutant Ras genes were among the first oncogenes identified for their ability to transform cells to a cancerous phenotype. The highest incidences of Ras mutations are found in cancers of the pancreas (80%) and colon (50%) (Duursma et al. 2003; Minamoto et al. 2000). Mutations in one of three genes (H, N or K-Ras) encoding Ras proteins are associated with increased cell proliferation and are found in an estimated 30%–40% of all human cancers. The Ras-Raf pathway is used by cells to transmit signals from the cell surface to the nucleus. Such signals direct cells to divide, differentiate or even undergo apoptosis. The Ras protein usually behaves as a relay switch in the signal pathway that triggers the cell to divide. In the absence of stimulus, the Ras protein remains in the “off” position. In response to external stimuli, Ras activates the cell-signaling pathways. A mutated Ras protein, however, is like a switch stuck in the “on” position. It continuously signals the cell to divide when the cycle should have actually been turned off (Gibbs et al. 1996). Irrespective of whether it is normal or a mutant, the newly formed Ras molecules are functionally immature. Precursor Ras genes must undergo biochemical modification called farnesylation to become mature, active versions. The farnesylation of the Ras proteins enable it to attach to the inner surface of the cells outer membrane where they can interact with other cellular proteins and stimulate cell growth. Statins block the HMG-COA reductase enzyme, which depletes cells of farnesyl pyrophosphate. Although the levels of total Ras do not decrease, the amount of farnesylated Ras decreases, which in turn, leads to reduced cell division (Hohl et al. 1995).

Prenylation also plays an important role in stimulating the development, angiogenesis and metastasis of cells in response to changes in the extracellular environment. Rho GTPases play a central role in diverse biological processes, such as actin cytoskeleton organization, microtubule dynamics, gene transcription, oncogenic transformation, cell cycle progression, adhesion and malignant transformation (Hall, 1998; Linda and Crislyn, 1997). The Rho family of proteins is made up of three subfamilies, Rho, Rac and Cdc42. The Rho subfamily regulates the formation of stress fibers and focal adhesions within cells, while the Rac subfamily regulates the formation of lamellipodia and membrane ruffling, whereas the Cdc42 subfamily regulates formation of filopodia (Jaffe and Hall, 2005; Burridge and Wennerberg, 2004). Elevated levels of Rho-GTP have been observed in several human cancers. A decrease in Rho activities has a negative impact on cell growth, suggesting that active Rho promotes tumor cell proliferation (Khosravi-Far et al. 1995). Rho proteins cycle between an active GTP-bound state and an inactive GDP-bound state (Etienne-Manneville and Hall, 2002). Their activation state is controlled by regulatory proteins, such as guanine exchange factors (GEFs), which catalyze the exchange of GDP for GTP, thereby activating Rho (Etienne-Manneville and Hall, 2002). In contrast, Guanine dissociation inhibitors (GDIs), which inhibit the release of GDP and GTPase activating proteins (GAPs), increases the rate at which Rho hydrolyzes GTP keeps the Rho inactivated. Rho-associated serine-threonine protein kinase (ROCK), one of the best characterized downstream effectors of Rho, is activated when it selectively binds to the active GTP-bound form of Rho. Activated ROCK interacts with the actin cytoskeleton to promote stress-fiber formation and assembly of focal contacts (Kamai et al. 2003). Cancer cell migration is central to the process of metastasis. This involves the rearrangement of the actin cytoskeleton. Since the Rho/ROCK pathway takes part in cancer progression by regulating actin cytoskeleton reorganization and cell migration, statins by blocking Rho prenylation can inhibit tumor growth and metastasis.

The nuclear architecture is defined by a specialized cytoskeleton, called the nuclear lamina. Nuclear lamina is formed by the type V intermediate filament proteins, the lamins (Aebi et al. 1986). Lamin B, along with Lamin A and C, together form a complex meshwork of tetragonally organized 10 nm filaments underneath the inner nuclear membrane (Stuurman et al. 1998). B-type lamins are ubiquitously expressed during development and every cell expresses at least one type of lamin B. There are two B-type lamins, B1 and B2, expressed from different genes. B type lamins are essential, while the A-type lamins are believed to be non-essential and are only expressed in differentiated cells (Foisner, 2001). B-type lamins contain a stable C-terminal farnesyl modification, which is important for targeting and anchoring the protein to the inner nuclear membrane (Firmbach-Kraft, and Stick, 1995). The first direct connection between lamin B and chromatin remodeling has been demonstrated by the discovery of the inner nuclear membrane protein, RING finger-binding protein (RFBP) (Mansharamani et al. 2001). RFBP binds to lamin B and interacts directly with the RUSH protein to remodel chromatin complexes. The karyoskeleton formed by the lamin proteins serves to organize protein complexes within the nucleus and their interactions with chromatin, as well as providing structural support to the nucleus (Gruenbaum et al. 2005; Taddei et al. 2004). In doing so, lamins are involved in a range of nuclear functions, including regulation of gene expression and DNA replication, although the molecular details of these functions are still to be elucidated. Lamin B depletion by RNA interference in *C. elegans* is lethal and associated with a number of phenotypic changes, such as irregularly organized nuclear pore complexes, irregularities in nuclear shape and defects in chromosomal organization and segregation (Liu et al. 2000). A recent study shows that lamin B functions in mitotic spindle assembly through a process that involves the GTP bound form of the small GTPase Ran. Lamin B is essential for the formation of the mitotic matrix that binds together a number of spindle assembly factors. Lamin B, therefore, by forming the mitotic spindle matrix, promotes microtubule assembly and organization. Blocking the prenylation of lamin B with drugs like statins will prevent their entry into nucleus and ability of the cancer cells to divide.

**Figure 2:**
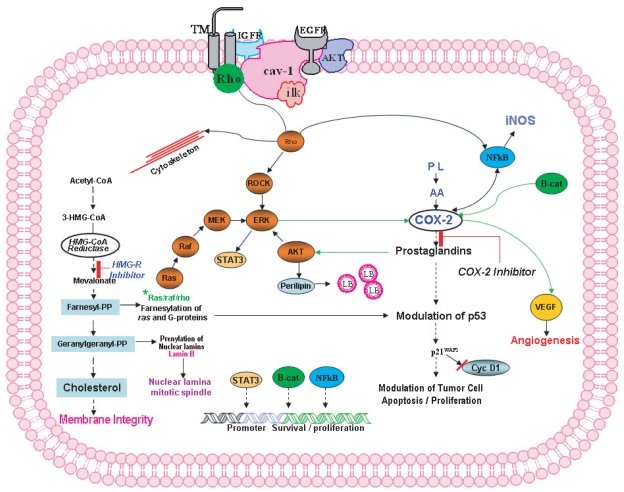
COX-2 and HMG-COA reductase pathway and its associated signaling molecules HMG-R inhibitors block the conversion of 3-HMG-CoA to mevalonate, preventing the formation of Farnesyl-PP and Geranylgeranyl-PP and subsequently, prenylation of Ras, Raf, Rho and lamin B. HMG-R inhibitors also block cholesterol, thereby disrupting Cav-1 associated signaling and lipid bodies. COX-2 activates β-Catenin, AKT and subsequently STAT3 and NFκB. STAT3, β-Catenin and NFκB then translocate to the nucleus and transcribe genes involved in survival and proliferation. COX-2 also promotes lipid body formation. **Abbreviations:** NFκB: nuclear factor κB; Cyc D1: Cyclin D1; B-Cat: β-catenin; ERK: extracellular regulated kinase; AKT: also known as protein kinase B (PKB); STAT3: signal trandsducer and activator of transcription; Cav-1: caveolin-1; EGFR: epidermal growth factor receptor; IGFR: insulin growth factor receptor; TM: transmembrane protein; LB: lipid bodies; PL: phospho lipids; AA: arachidonic acid; COX-2: cyclooxygenase 2; VEGF: vascular endothelial growth factor; iNOS: inducible nitric oxide synthase; ilk: integrin linked kinase; ROCK: Rho kinase: MEK: MAP kinase.

Although statins have a proven effect of reducing serum LDL concentration, several lines of evidence suggest that statins exhibit some interesting effects that cannot be explained by lowering of the LDL levels alone. While the effect of statins on caveolae disruption and caveolin-1 function may be related to its ability to block cholesterol formation, the effect of statins on proteins like lamin B, Ras and Rho is due to the unavailability of cholesterol biosynthetic pathway intermediates called isorenoids. HMG-CoA reductase inhibitors, such as statins, block the first step in the cholesterol biosynthetic pathway. Therefore, by using statins, the end product cholesterol, as well as the production of several intermediates in the cholesterol biosynthetic pathway is inhibited. As the synthesis of mevalonate by the enzyme HMG-CoA reductase is a committed step in both, cholesterol and prenyl lipid biosynthesis pathways, the use of HMG-CoA reductase inhibitors, such as the statins, could be used to inhibit prenylation. Ras, both mutant and wild type, must be farnesylated for proper processing, subcellular localization and biological activity. Interestingly, statins ability to block the prenylation of several important proteins, like Ras and Rho is important in regulating the growth of cancer cells. The pleiotropic effects of statins as discussed earlier, therefore, represent an area of great interest in the prevention and therapy of cancer. The schematic representation of COX-2 and HMG-CoA reductase signaling mechanism and the cross-talk between these two pathways is shown in Figure 2.

## Conclusion

In principle, targeting a single gene should be sufficient to induce apoptosis or decrease proliferation effectively in cancer cells. However, considering that several genes belonging to different metabolic pathways are upregulated, the traditional single-drug, single-target approach should be replaced by the multi-drug, multiple-target approach. The aim of the present review was to provide the knowledge of how a combination of COX-2 inhibitors and HMG-CoA reductase inhibitors can be used to block various signaling cascades within the context of COX-2 and HMG-CoA reductase pathways. With regard to COX-2 inhibitors, recent evidence indicates that patients treated with selective COX-2 inhibitors exhibit slightly increased risk of cardiovascular problems such as heart attacks and strokes. COX-2 reduces prostacyclin formation by tipping the balance of prostacyclin/thromboxane in favor of thromboxane, a prothrombotic eicosanoid. The increased levels of thromboxane, accompanied by a decrease in prostacyclin can lead to the development of thrombotic cardiovascular events (Bing and Lomnicka, 2002). This may be prevented by the addition of a nitric oxide donor to the parent compound such as aspirin or pravastatin through chemical spacers, such as aliphatic, aromatic, or a heterocyclic chain. This approach has led to the synthesis of several new drugs such as NO-aspirin, NO-naproxen and more recently NO-pravastatin, which are not only more effective than the parent compound, but also exhibit a better safety profile. The possibilities described above makes it an attractive option to test several anti-cancer drug combinations.

## Figures and Tables

**Table 1:** List of prenylated proteins.

Protein	Type of modification
LaminB (Mitotic spindle)	Farnesylation
H-Ras (Signal transduction)	Farnesylation
K-Ras (Signal transduction)	Farnesylation/Geranylgeranylation
Cdc42 (Cell cycle)	Geranylgeranylation
Rac (Signal transduction)	Geranylgeranylation
Rab (Vesicle transport)	Geranylgeranylation
Rho (Signal transduction/Cytoskeleton)	Geranylgeranylation
γ-subunit of G proteins	Geranylgeranylation
Rhodopsin kinase (Visual cycle)	Farnesylation
